# RNArchitects: how hnRNPs shape neuronal landscapes

**DOI:** 10.1093/brain/awaf448

**Published:** 2025-12-01

**Authors:** Afonso Lourinho, Marta Silva, Emanuel Tahiri, Ana Pinto, Beatriz Caniceiro, Irina Moreira, Carlos B Duarte, Rui O Costa

**Affiliations:** CNC-UC - Center for Neuroscience and Cell Biology, University of Coimbra, 3004-504 Coimbra, Portugal; CIBB - Center for Innovative Biomedicine and Biotechnology, University of Coimbra, 3000-548 Coimbra, Portugal; CNC-UC - Center for Neuroscience and Cell Biology, University of Coimbra, 3004-504 Coimbra, Portugal; CIBB - Center for Innovative Biomedicine and Biotechnology, University of Coimbra, 3000-548 Coimbra, Portugal; CNC-UC - Center for Neuroscience and Cell Biology, University of Coimbra, 3004-504 Coimbra, Portugal; CIBB - Center for Innovative Biomedicine and Biotechnology, University of Coimbra, 3000-548 Coimbra, Portugal; Institute for Interdisciplinary Research, University of Coimbra, 3030-789 Coimbra, Portugal; PDBEB - Doctoral Programme in Experimental Biology and Biomedicine, University of Coimbra, 3004-504 Coimbra, Portugal; CNC-UC - Center for Neuroscience and Cell Biology, University of Coimbra, 3004-504 Coimbra, Portugal; CIBB - Center for Innovative Biomedicine and Biotechnology, University of Coimbra, 3000-548 Coimbra, Portugal; CNC-UC - Center for Neuroscience and Cell Biology, University of Coimbra, 3004-504 Coimbra, Portugal; CIBB - Center for Innovative Biomedicine and Biotechnology, University of Coimbra, 3000-548 Coimbra, Portugal; CNC-UC - Center for Neuroscience and Cell Biology, University of Coimbra, 3004-504 Coimbra, Portugal; CIBB - Center for Innovative Biomedicine and Biotechnology, University of Coimbra, 3000-548 Coimbra, Portugal; CNC-UC - Center for Neuroscience and Cell Biology, University of Coimbra, 3004-504 Coimbra, Portugal; CIBB - Center for Innovative Biomedicine and Biotechnology, University of Coimbra, 3000-548 Coimbra, Portugal; Department of Life Sciences, University of Coimbra, 3000-456 Coimbra, Portugal; CNC-UC - Center for Neuroscience and Cell Biology, University of Coimbra, 3004-504 Coimbra, Portugal; CIBB - Center for Innovative Biomedicine and Biotechnology, University of Coimbra, 3000-548 Coimbra, Portugal; Institute for Interdisciplinary Research, University of Coimbra, 3030-789 Coimbra, Portugal

**Keywords:** RNA metabolism, heterogeneous nuclear ribonucleoproteins, neuronal development, neurodegenerative diseases, neurodevelopmental disorders, RNA binding proteins

## Abstract

Neurons rely on finely tuned RNA regulatory mechanisms to sustain their specialized functions, with heterogeneous nuclear ribonucleoproteins (hnRNPs) emerging as key regulators of these processes. hnRNPs exert multilayered control over synaptic plasticity, axonal function, and neurodevelopmental gene expression by dynamically coordinating mRNA splicing, stability, transport and local translation. Given their pivotal role in neuronal RNA metabolism, recent discoveries have highlighted how hnRNP dysfunction drives pathological RNA dysregulation across a spectrum of neurological disorders.

This review provides insights into hnRNP-mediated RNA regulation in the brain, examines their contributions to neurological diseases, and explores how targeting these proteins could pave the way for novel therapeutic strategies to preserve neuronal integrity across diverse neurological conditions.

## Teaser

Heterogeneous nuclear ribonucleoproteins (hnRNPs) regulate neuronal mRNA splicing, stability, transport, and translation, shaping synaptic plasticity and axonal function. Lourinho *et al.* review how hnRNP dysfunction contributes to neurological disorders and how targeting these proteins may offer new therapeutic opportunities.

## Heterogeneous nuclear ribonucleoproteins

In the intricate world of eukaryotic gene expression, the maturation of protein-coding transcripts is a tightly regulated, multi-step process that begins with the synthesis of precursor mRNAs (pre-mRNAs) by RNA polymerase II. These initial transcripts, historically referred to as heterogeneous nuclear RNAs, undergo extensive post-transcriptional processing to generate functional mRNAs that can be exported from the nucleus and translated.^1^ A central component of this maturation process is the association with RNA-binding proteins, which coordinate key steps such as splicing, polyadenylation, and transport. Among these, the heterogeneous nuclear ribonucleoproteins (hnRNPs) represent a diverse and highly abundant family of RNA-binding proteins that associate with nascent pre-mRNAs from the earliest stages of transcription.^2^ hnRNPs do not merely chaperone pre-mRNAs during their maturation; together with other RNA-binding proteins, they actively shape RNA metabolism, dynamically associating and dissociating with transcripts to fine-tune their processing, stability, and ultimate functionality.^3,4^ At the mechanistic level, hnRNPs regulate alternative splicing by recognizing specific RNA motifs that influence splice-site selection and spliceosome assembly. Their interactions are further modulated by RNA chemical modifications, such as *N*^6^-methyladenosine (m^6^A), pseudouridine (Ψ), m^1^A and m^5^C, which can alter RNA structure and, consequently, hnRNP binding dynamics.^5^ Broader analyses highlight hnRNPs as central components of the wider RNA-binding protein network controlling RNA fate in health and disease.^6^

While hnRNPs are essential regulators of RNA metabolism in all cells, their roles in the nervous system are particularly profound. Neurons rely on highly specialized RNA regulatory mechanisms to maintain their function, morphology and plasticity, processes that demand precise spatial and temporal control of gene expression. hnRNPs are deeply embedded in these regulatory networks, modulating several aspects of RNA metabolism to sustain neuronal integrity.^7^ However, when hnRNP function is perturbed, the consequences are profound. Indeed, dysregulated hnRNP activity has been implicated in a spectrum of neurological disorders, ranging from neurodevelopmental syndromes to neurodegenerative diseases.

This review synthesizes our current understanding of the hnRNP family, emphasizing its essential roles in neuronal physiology and their contributions to neurological diseases. By dissecting the diverse functions of hnRNPs in neurons, we aim to provide new insights into the molecular underpinnings of neurological disorders and explore potential therapeutic avenues that could emerge from targeting this crucial family of RNA-binding proteins.

### The heterogeneous nuclear ribonucleoprotein family

**Figure 1:**
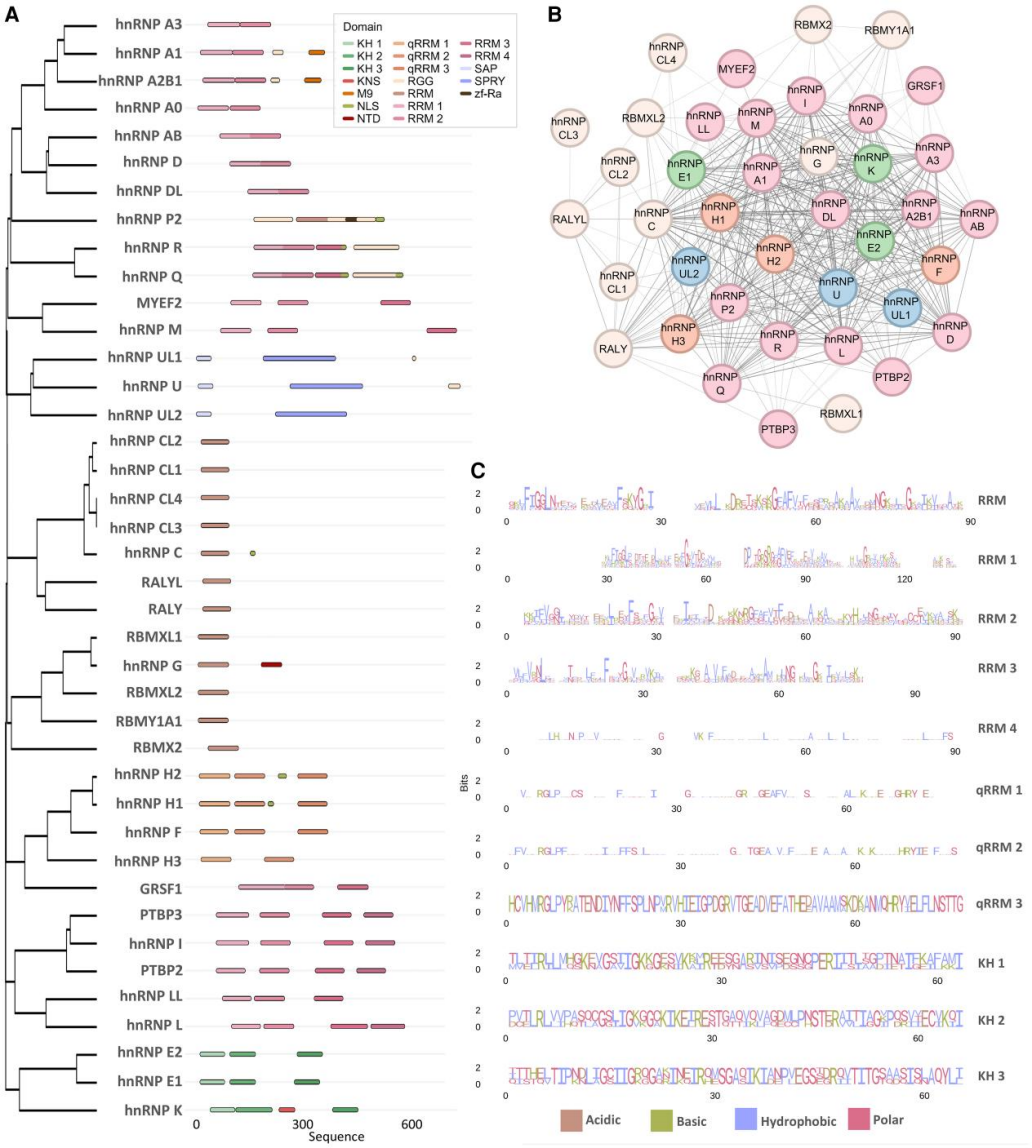
**Domain organization, phylogenetic relationships, interaction network and sequence conservation of the heterogeneous nuclear ribonucleoprotein family.** Heterogeneous nuclear ribonucleoproteins (hnRNPs) play essential roles in RNA processing, transport and regulation. Evolution has shaped their diversity in domain composition, interaction networks and functions, establishing them as central modulators of RNA metabolism. (**A**) The phylogenetic tree of hnRNPs based on sequence similarity reveals distinct subfamilies, with domains mapped along the amino acid sequence to highlight their structural organization. Conserved domains are colour-coded according to function, and the tree and schematics were generated using the ggtree^11^ and ggplot2^12^ packages in R (https://www.R-project.org/). (**B**) hnRNPs form extensive protein–protein interaction networks, frequently associating with other hnRNP family members. The interaction network, generated using the STRING database^13^ with a minimum required interaction score set to medium confidence (0.400), and visualized in Cytoscape,^14^ is colour-coded by the most prominent domain within each protein: blue for SPRY, green for KH 1/2/3, orange for qRRM 1/2/3, pink for RRM 1/2/3/4 and beige for RRM. (**C**) Many hnRNPs contain highly conserved RNA-binding motifs, which are critical for their regulatory roles in RNA metabolism. Sequence conservation logos of major hnRNP domains highlight specific conserved residues, with amino acids colour-coded by physicochemical properties: acidic (brown), basic (blue), hydrophobic (green) and polar (pink). Logos were generated using the ggseqlogo package in R.^15^ KH 1 = K-homology domain 1; KH 2 = K-homology domain 2; KH 3 = K-homology domain 3; KNS = hnRNP K nuclear shuttling domain; NLS = nuclear localization signal; PY-NLS = proline-tyrosine nuclear localization signal; NTD = nascent transcripts targeting domain; qRRM 1 = quasi-RNA recognition motif 1; qRRM 2 = quasi-RNA recognition motif 2; qRRM 3 = quasi-RNA recognition motif 3; RGG = arginine–glycine–glycine repeating domain; RRM = RNA recognition motif; RRM 1 = RNA recognition motif 1; RRM 2 = RNA recognition motif 2; RRM 3 = RNA recognition motif 3; RRM 4 = RNA recognition motif 4; SAP = SAF-A/B (scaffold attachment factor A/B), ACINUS (apoptotic chromatin condensation inducer in the nucleus) and PIAS (protein inhibitor of activated signal transducer and activator of transcription) domain; SPRY = SPla and RYanodine receptor domain; zf-Ra = zinc finger of RAN-binding protein domain.

The hnRNP family is composed of 20 major canonical sub-families, each containing multiple paralogues and, in some cases, more distantly related proteins.^8^ These sub-families, designated alphabetically from hnRNP A1 to U, encompass proteins with molecular weights ranging from 34 to 120 kDa (Fig. 1A).^1,9,10^ Despite their differences, hnRNPs frequently interact (Fig. 1B) and co-assemble within the same complexes, suggesting they share RNA-binding properties and overlapping cellular functions.^16^

Heterogeneous nuclear RNPs are modular and composed of multiple structural domains connected by variable linker regions.^2^ Structural analyses have revealed that these proteins typically contain one or more RNA-binding domains, sequence motifs that regulate their intracellular localization and redistribution, and auxiliary domains that contribute to protein–protein interactions or RNA processing functions.^17^ The most prevalent RNA-binding domains in hnRNPs include the RNA-recognition motif (RRM), quasi-RRM (qRRM), K homology (KH) domain and arginine-glycine-glycine (RGG) repeating domain, all of which facilitate interactions with a broad spectrum of RNA targets and govern the nucleic acid binding capabilities of hnRNPs^18^ (Fig. 1C). Intrinsically disordered regions within hnRNPs also contribute to RNA binding, enabling these proteins to engage in a wide range of RNA regulatory processes through both specific and non-specific interactions.^19,20^

Beyond RNA binding, several domains govern the intracellular localization of hnRNPs, ensuring their proper distribution and function. These are among the most abundant nuclear proteins, rivalling histones in prevalence, yet their localization is highly dynamic.^17^ While some hnRNPs remain nuclear, others shuttle between the nucleus and cytoplasm, an essential process for their roles in regulating RNA metabolism and gene expression. This distribution is controlled by specific sequence motifs: nuclear localization signals (NLS) mediate nuclear retention, whereas nucleocytoplasmic shuttling (NS) domains enable bidirectional transport.^18^ For instance, hnRNP C is retained in the nucleus by a classical NLS, while hnRNP A1 relies on a non-classical proline-tyrosine NLS (PY-NLS), also known as the M9 domain, for bidirectional transport.^21-23^ Although primarily nuclear, hnRNP K can shuttle via its KNS (hnRNP K nuclear shuttling) domain, which facilitates its compartment-specific functions.^24^ Similarly, hnRNP G harbours a nascent transcripts targeting domain (NTD) linked to its splicing regulatory role and nuclear localization.^25^ hnRNPs also contain a range of highly variable auxiliary domains, such as acid-rich, proline-rich and glycine-rich regions, which provide further functional diversity to the hnRNP family by regulating protein–protein interactions and subcellular localization.^8^

As multifunctional proteins, hnRNPs are involved in a broad range of cellular processes, with expression patterns that vary widely across tissues. Their stoichiometry is dynamic and cell-type specific, highlighting their critical role as global regulators of RNA metabolism and their capacity to fine-tune gene expression in a cell-specific manner.^26^ Together with other RNA-binding proteins, hnRNPs orchestrate multiple stages of pre-mRNA processing, including transcription and splicing, as well as the subsequent export, localization and translation of these transcripts within the cell.^27^ Furthermore, hnRNPs are implicated in the regulation of mRNA stability, RNA silencing, DNA repair, telomere biogenesis and the maintenance of transcriptome integrity.^28^

Although hnRNP paralogues often exhibit overlapping functions, as exemplified by members of the hnRNP F/H subfamily, including hnRNP H2, their distinct binding affinities and dynamic localization patterns render them non-redundant. Whether retained in the nucleus or dynamically shuttling between compartments, these proteins form complex, cooperative networks with other molecular partners, and their distinct binding affinities likely guide them to specific nucleic acid and protein targets.^2^ As previously acknowledged, hnRNPs fulfil both ‘generalised roles as RNA packaging proteins, and specialised roles that are dependent on specific RNA–protein or protein–protein interactions’.^2^

### Heterogeneous nuclear ribonucleoproteins as key regulators of neuronal function

Neurons achieve remarkable functional diversity and adaptability through intricate layers of post-transcriptional gene regulation. Among the RNA-binding proteins that orchestrate these processes, hnRNPs have emerged as dynamic regulators of mRNA fate, shaping neuronal activity, morphology, and plasticity. This section outlines several studies demonstrating how specific neuronal mechanisms are regulated at the level of hnRNP activity, emphasizing their essential roles in maintaining brain function and development.

#### Dendritic protein synthesis and synaptic transmission

**Figure 2:**
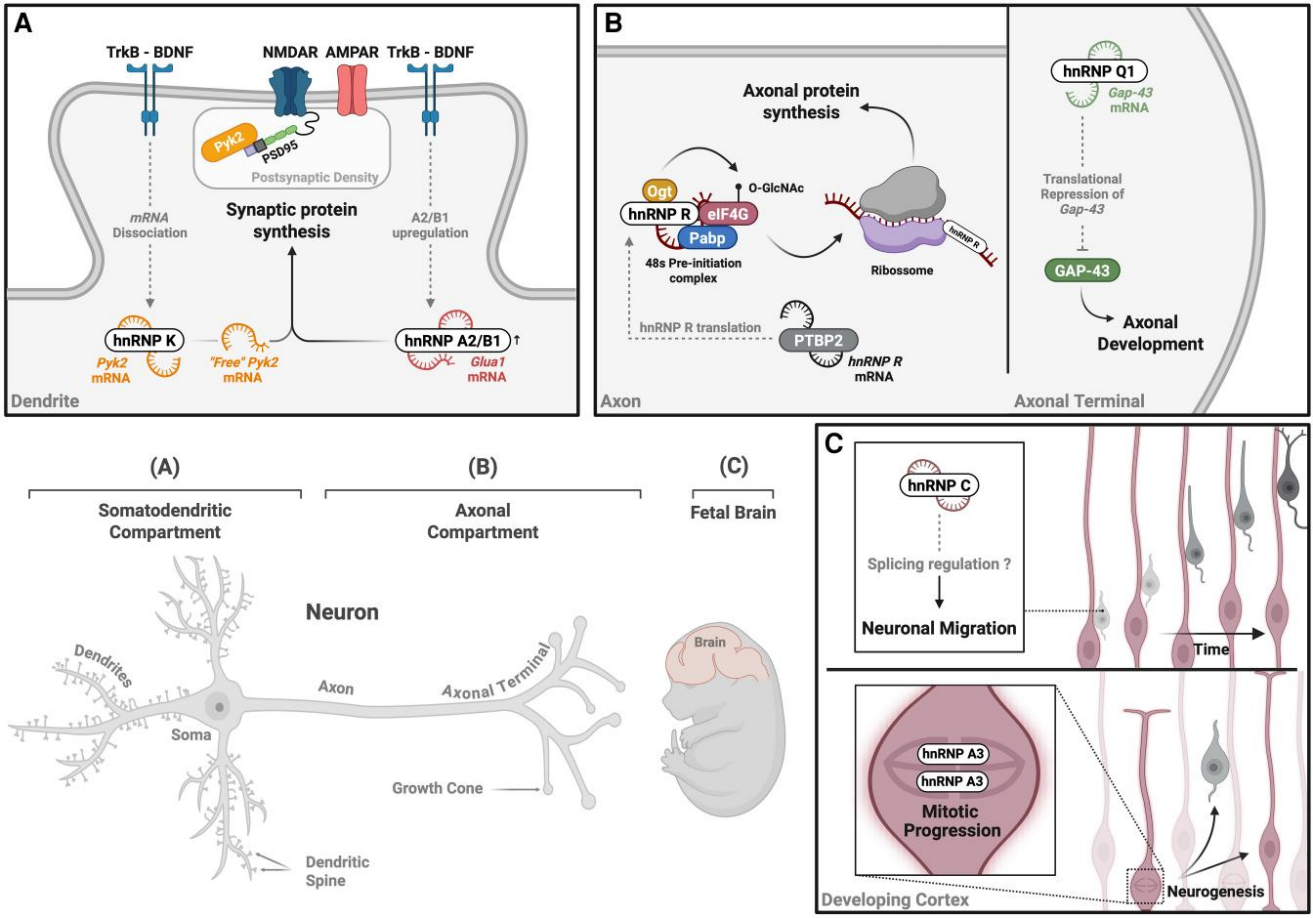
**Neuronal roles of the heterogeneous nuclear ribonucleoprotein family.** Heterogeneous nuclear ribonucleoproteins (hnRNPs) orchestrate RNA metabolism within distinct neuronal compartments, enabling precise control of gene expression in the brain. This intricate regulation supports both the rapid synaptic modifications of mature neurons and the fundamental processes guiding early neurodevelopment. (**A**) In dendrites, hnRNP K and hnRNP A2/B1 mediate the localized fate of mRNAs. hnRNP K facilitates the dendritic transport of specific neuronal transcripts and, in response to BDNF stimulation, triggers their release for on-site translation, thereby rapidly modulating the composition and function of key synaptic components such as NMDA receptors.^31,34-36^ Concurrently, hnRNP A2/B1 directs A2RE-containing mRNAs to synaptic sites, ensuring the timely synthesis of essential synaptic components that reinforce both synaptic transmission and long-term potentiation.^34,37,38^ (**B**) Within axons, hnRNP R is locally synthesized under the regulation of PTBP2, supporting the production of proteins vital for maintaining axonal structure and function via interactions with the translation initiation complex.^39,40^ In parallel, hnRNP Q1 exerts translational repression on GAP-43, a critical regulator of axonal development.^41-43^ These targeted mechanisms ensure that axonal protein expression is spatially and temporally fine-tuned, preserving axonal integrity and function. (**C**) Emerging evidence further implicates hnRNPs in neurodevelopmental processes. hnRNP C modulates the alternative splicing of genes critical for neurodevelopment, thereby influencing neuronal morphology and the migration of cortical neurons, although the mechanistic link between these processes remains to be fully established.^44^ In a non-canonical role, hnRNP A3 interacts with cohesin complexes to regulate mitotic progression in neural progenitors, ensuring accurate chromosome segregation during neurogenesis.^45^ Created in BioRender. Costa, R. (2026) https://BioRender.com/idisog4. AMPAR = α-amino-3-hydroxy-5-methyl-4-isoxazole propionic acid receptor; BDNF = brain-derived neurotrophic factor; EIF4G = human eukaryotic translation initiation factor 4G; GAP-43 = growth-associated protein 43; GluA1 = glutamate ionotropic receptor AMPA type subunit 1; NMDAR = *N*-methyl-D-aspartate receptor; Ogt = *O*-linked β-*N*-acetylglucosamine (O-GlcNAc) transferase; PABP = polyadenylate-binding protein; PSD-95 = postsynaptic density protein 95; PTBP2 = polypyrimidine tract binding protein 2; Pyk2 = proline-rich tyrosine kinase 2; TrkB = tropomyosin receptor kinase B.

Synapse development and plasticity rely on local protein synthesis at the synapse, which requires the transport of mRNAs from the nucleus to these specific regions.^7^ Indeed, local protein synthesis has been shown to play critical roles in several forms of synaptic plasticity, including brain-derived neurotrophic factor (BDNF)-mediated long-term potentiation (LTP).^29-31^ Several studies have highlighted hnRNP K as an essential factor in various key neuronal processes, particularly in the regulation of LTP and neuronal RNA transport.^32-35^ Notably, neuronal activity has been shown to drive hnRNP K accumulation in dendrites via a BDNF-dependent mechanism (Fig. 2A). Microarray analysis identified numerous transcripts that co-immunoprecipitated with hnRNP K in cultured rat hippocampal neurons. At synaptic sites, BDNF stimulates the release of hnRNP K-associated transcripts, which encode key synaptic proteins.^31,34^ Building on these findings, recent research confirmed that BDNF promotes the dissociation of *Pyk2* mRNA from hnRNP K, leading to the local synthesis of Pyk2 at the synapse.^35^ Pyk2, a non-receptor tyrosine kinase, plays a pivotal role in the NMDA receptor complex, where it modulates NMDAR activity and function.^36^ Interestingly, BDNF stimulation was shown to enhance the synaptic expression of GluN2B-containing NMDAR by a mechanism dependent on Pyk2 expression, showing that hnRNP K orchestrates BDNF-induced changes in NMDAR composition and function.^35^ Interestingly, hnRNP K has also been recently shown to regulate BDNF expression, further highlighting its role in synaptic modulation.^46^

In addition to hnRNP K, hnRNP A2/B1 is well-established as a key RNA-binding protein involved in the trafficking of mRNAs essential for synapse formation and plasticity (Fig. 2A). hnRNP A2/B1 specifically recognizes the hnRNP A2 response element (A2RE) present in mRNA. In neurons, this protein is involved in the dendritic delivery of A2RE-containing transcripts that encode proteins involved in synapse formation and plasticity, such as α-CaMKII, neurogranin and Arc.^37^ Neuronal activity and BDNF treatment elevate hnRNP A2/B1 protein levels in both the cell body and dendritic compartments, leading to its accumulation at synaptic sites.^47^ Recent studies further highlight that BDNF-induced local translation of GluA1 is regulated by hnRNP A2/B1, emphasizing its role in localized mRNA processing and its potential regulatory function in BDNF-mediated synaptic plasticity.^38^ Additionally, hnRNP A2/B1 has been identified as a key regulator of neuronal proteostasis, mediating the dendritic localization of heat shock protein family A member 8 (HSPA8) mRNA.^48^ This further underscores its critical role in neuronal RNA transport and local protein synthesis.

In summary, these studies highlight the pivotal roles of hnRNP K and A2/B1 in regulating dendritic protein synthesis and synaptic transmission. By orchestrating mRNA trafficking and local translation, these RNA-binding proteins ensure the precise expression of key synaptic proteins, ultimately influencing neuronal communication and the molecular mechanisms that underlie synaptic plasticity. Given the structural and functional diversity of hnRNPs, other family members may also contribute to these dendritic and synaptic regulatory processes in ways yet to be uncovered.

#### Axonal growth, transport and function

Axons, like dendrites, are rich in RNA-binding proteins, including many hnRNP family members that facilitate the spatiotemporal regulation of RNA metabolism necessary for axonal function.^49^ These proteins play key roles in regulating mRNA transport and local translation, both of which are crucial for proper axonal growth and development. For instance, hnRNP Q1 is known to regulate axonal development by repressing *GAP43* mRNA translation, thereby inhibiting the expression of GAP43, a protein crucial for actin dynamics and axonal growth^41-43^ (Fig. 2B). hnRNP Q1 also regulates the post-transcriptional processing of several other mRNAs that encode key regulators of cytoskeletal dynamics, including components of the Cdc42/N-WASP/Arp2/3 complex and RhoA.^50,51^ Similarly, hnRNP R modulates axonal growth and maintenance in motor neurons by interacting with the noncoding RNA 7SK, while also controlling the transport and integrity of axonal mitochondria.^52,53^ Furthermore, recent studies show that hnRNP R regulates axonal protein synthesis by interacting with the translational machinery to control the expression of cytoskeletal and synaptic proteins. Loss of hnRNP R impairs motor function and neuromuscular junction innervation *in vivo*, further supporting its essential role in regulating axonal function and development.^39^ Intriguingly, hnRNP R itself is locally synthesized from axonal *HNRNPR* transcripts through a process dependent on the polypyrimidine tract binding protein 2 (PTBP2), highlighting a cross-regulatory network among hnRNPs that fine-tune axonal protein synthesis^40^ (Fig. 2B).

hnRNP A/B also controls local mRNA translation during axon development. In olfactory sensory neurons, hnRNP A/B regulates mRNAs encoding cell adhesion molecules such as protocadherin alpha (PCDHA) and neural cell adhesion molecule 2 (NCAM2), which are essential for axonal targeting and maturation. hnRNP A/B depletion results in improper targeting of olfactory sensory neuron axons to the olfactory bulb, along with decreased levels of these adhesion molecules at the axon terminals.^54^

Taken together, these findings highlight the diverse and interconnected roles of hnRNP family members in the regulation of axonal mRNA metabolism and translation. Importantly, these regulatory processes are crucial not only for axonal development and function but also for maintaining axonal integrity. For instance, PTBP1 (hnRNP I) is upregulated in axons after peripheral nerve injury, where it regulates mRNAs involved in injury responses and nerve regeneration.^55^ Thus, hnRNPs have emerged as critical modulators of axonal biology, constituting a complex and dynamic network that ensures the maintenance of axonal functionality across various neuronal contexts.

#### Neurodevelopmental mechanisms

The regulation of various neurodevelopmental processes is linked to hnRNPs.^7^ For instance, hnRNP M was first identified as a regulator of dopamine D2 receptor (D2R) pre-mRNA splicing, where it favours the production of the presynaptic short form (D2S) isoform.^56,57^ More recently, hnRNP M has been implicated in broader neurodevelopmental processes, including learning and memory, as its disruption in the hippocampus leads to altered dendritic spine density and cognitive deficits.^58^ Additionally, hnRNP M regulates key synaptic proteins such as synaptophysin and postsynaptic density protein 95 (PSD95) by stabilizing their mRNAs, reinforcing its essential role in neuronal development.^58^

As a crucial regulator of neurodevelopment, hnRNP U orchestrates gene expression, splicing and chromatin dynamics essential for brain maturation. *Hnrnpu* haploinsufficiency in mice disrupts key transcriptional programs in hippocampal and neocortical cells, while conditional knockout of hnRNP U in the developing cerebral cortex leads to neural progenitor cell death and dysregulation of neuronal gene expression and splicing.^59^  ^,60^ Recent studies using human-derived models further demonstrate that hnRNP U deficiency induces widespread transcriptomic alterations, highlighting its central role in coordinating neurodevelopmental transcriptional programs.^61,62^ Mechanistically, hnRNP U directly binds mRNAs encoding essential neurodevelopmental proteins involved in neurite formation, many of which exhibit consistent transcriptional and splicing dysregulation in both human and mouse models of *HNRNPU* deficiency.^63^ Beyond RNA processing, hnRNP U also modulates neuronal RNA stability and translation, while influencing DNA methylation during neural differentiation, collectively shaping gene expression programs vital for brain development.^63^

hnRNP C has also been implicated in critical neurodevelopmental processes.^44^ In human cell lines, hnRNP C knockdown leads to alterations in the alternative splicing of genes associated with intellectual disability. Further *in vivo* experiments using *in utero* electroporation in mice revealed that hnRNP C knockdown in cells destined to form the somatosensory cortex leads to aberrant neuronal migration to the cortical plate. Remarkably, overexpression of wild-type hnRNP C mirrors the effects of its depletion, suggesting that a precise balance of hnRNP C is essential for proper cortical development^44^ (Fig. 2C). Lastly, hnRNP A3 has been identified as another key player in neural development. It is expressed early during brain development, particularly in cortical neural progenitors of both mice and humans. Deletion of hnRNP A3 in mice results in prolonged mitosis and a reduced production of deep-layer neurons, a phenotype also observed when *HNRNPA3* is downregulated in cultured human cerebral organoids^45^ (Fig. 2C). Overall, these recent findings place hnRNPs at the heart of neurodevelopmental regulation, with aberrations in their function linked to alterations in RNA metabolism and improper brain formation.

The increasing recognition of the hnRNP family as a critical regulator of both neuronal function and development marks a paradigm shift in our understanding of brain biology. From the well-established roles of hnRNP A2/B1 and U to the emerging functions of less characterized members like hnRNP H2, these multifunctional RNA-binding proteins orchestrate a diverse array of processes vital for maintaining neuronal integrity and adaptability. Notably, their contributions extend from controlling localized protein synthesis at synapses and axons to governing the alternative splicing of transcripts essential for cortical development. Such findings not only highlight the hnRNP family as crucial mediators of neuronal development and function but also provide a window into how dysregulation of these processes may contribute to neurological disorders.

### Understanding neurodegenerative brain diseases through the lens of heterogeneous nuclear ribonucleoproteins

Long recognized as key players in cancer, extensive evidence links hnRNP dysregulation to tumorigenesis through diverse molecular mechanisms. Aberrant hnRNP expression and mutations in hnRNP genes have been observed across multiple cancer types.^64^ Functionally, hnRNPs influence key oncogenic pathways, including the IL6/JAK/STAT3 signalling cascade, DNA repair mechanisms and mitotic spindle assembly, processes frequently disrupted in tumorigenesis.^64^ While hnRNPs have also been linked to brain cancer, their impact extends beyond oncology, as growing research reveals their implication in other neurological disorders.^65,66^ Indeed, given the sensitivity of neurons to RNA-processing defects, hnRNP dysfunction has emerged as a key factor in various brain diseases.^67^ This section reviews studies that have uncovered the contributions of hnRNPs to neurodegenerative and neurodevelopmental disorders, focusing on their roles in disease onset and pathology.

#### Alzheimer’s disease

Alzheimer’s disease (AD) is a devastating neurodegenerative disorder characterized by the extracellular deposition of amyloid-β (Aβ) plaques and the intracellular accumulation of tau-containing neurofibrillary tangles.^68^ While these hallmark features have long defined AD pathology, emerging evidence identifies hnRNPs, particularly hnRNP A1, as critical regulators of both amyloidogenic and tau-related pathways.^69^

hnRNP A1 plays a pivotal role in the alternative splicing of amyloid precursor protein (APP), a precursor of Aβ peptides. By binding to Alu elements near exon 7, hnRNP A1 promotes exon skipping, leading to the production of APP695 mRNA, an isoform associated with reduced Aβ generation relative to the full-length APP transcript.^70,71^ Beyond its influence on APP, hnRNP A1 also governs the splicing of tau pre-mRNA, particularly the inclusion of exon 10, which determines the ratio of 3-repeat (3R) and 4-repeat (4R) tau isoforms.^72^ Knockdown of hnRNP A1 expression increases exon 10 inclusion in tau pre-mRNA, resulting in elevated 4R-Tau protein levels and a higher 4R-Tau/3R-Tau ratio, an imbalance that is a hallmark of the AD brain.^72-74^ Remarkably, the expression of hnRNP A1 is diminished in the brains of individuals with AD, as well as in 3xTg-AD mouse models and in neuroblastoma cell lines treated with Aβ oligomers.^75-77^ Experimental depletion of hnRNP A/B proteins *in vitro* induces synaptic and dendritic loss, while *in vivo* knockdown recapitulates core AD phenotypes in mice, including learning and memory impairments.^75^ As a key mediator of both APP and tau splicing, hnRNP A1 downregulation may represent a critical vulnerability in the AD brain, potentially disrupting neuronal homeostasis and accelerating disease progression. Mechanistically, recent studies reveal that Aβ oligomers downregulate hnRNP A1 via the activation of p-p38 MAPK signalling, suggesting a feedback loop wherein Aβ accumulation drives hnRNP A1 dysregulation, perpetuating amyloid pathology.^76^ However, transgenic mice with disease-associated mutations in APP display no changes in hnRNP A/B levels, indicating that its loss in AD may be independent of direct Aβ toxicity.^75^ Furthermore, hnRNP A1 shows significant subcellular mislocalization in AD and other tauopathies, which may also impair its cellular functions and contribute to disease progression.^78^

Contrary to hnRNP A1, hnRNP C stabilizes the APP precursor's mRNA and promotes APP translation by displacing fragile X messenger ribonucleoprotein (FMRP), relieving the translational block implemented by this other RNA-binding protein on the coding region element of *APP* mRNA.^79^ This suggests that increasing hnRNP C levels may promote Aβ secretion, which is interesting considering that elevated hnRNP C levels have been observed in animal models of AD and in hippocampal synaptosomes from patients with sporadic AD.^80^ In addition, human brain tissues from patients with AD revealed significantly increased levels of hnRNP C, while hnRNP A1 levels were significantly reduced, underscoring their opposing roles in AD pathology.^81^

Alterations in hnRNP levels extend well beyond hnRNP A1 and C, with proteomic studies reflecting widespread dysregulation within this protein family in brain tissues from patients with AD.^81^ Beyond expression changes, mislocalization of hnRNPs has emerged as a critical pathological feature in AD. For instance, hnRNP K is mislocalized to the cytoplasm in neurons of the dentate nucleus in AD post-mortem samples, while a recent study further showed that hnRNP A2/B1 is also mislocalized in brain tissue from patients with AD.^78,82^ Notably, induction of oligomeric tau (oTau) promotes the nuclear-to-cytoplasmic translocation of hnRNP A2/B1. Interestingly, the same authors demonstrated that the interaction between oTau and m^6^A-modified RNA, mediated by hnRNP A2/B1, triggers stress granule formation and exacerbates neurodegeneration in AD models, highlighting a mechanism by which altered hnRNP distribution may drive AD pathogenesis.^83^

Together, these findings highlight the critical role of hnRNPs in regulating the splicing of APP and tau, two key factors in AD pathology. The widespread expression changes and mislocalization of hnRNPs in the brains of patients with AD further emphasize their significance in disease progression, suggesting that targeting dysfunctional hnRNP pathways could offer promising new avenues for therapeutic interventions in AD.

#### Multiple sclerosis

Multiple sclerosis (MS) is an immune-mediated chronic inflammatory, demyelinating and neurodegenerative disease of the CNS. The pathological hallmark of MS is the formation of demyelinating lesions in the brain and spinal cord, which can be associated with neuro-axonal damage.^84^ While the mechanisms underlying MS are complex, disruptions in hnRNP proteins have emerged as key contributors to its pathogenesis.^69^

hnRNP A1, a critical regulator of nuclear morphology and stress granule formation, has emerged as a pivotal factor in the mechanisms underlying MS.^85-87^ In MS brains, hnRNP A1 undergoes mislocalization from the nucleus to the cytoplasm, where it aberrantly associates with stress granules, a hallmark of cellular stress.^88,89^ This cytoplasmic accumulation of hnRNP A1 is mirrored in experimental autoimmune encephalomyelitis (EAE), a widely used animal model of MS, where hnRNP A1 mislocalization and stress granule formation are consistently observed in spinal cord neurons of affected mice.^90^ While germline mutations in hnRNP A1 have not been implicated in MS, somatic mutations in hnRNP A1 were identified in progressive MS patients, which were found to alter hnRNP A1 cytoplasmic localization and to promote stress granule formation.^91^ Notably, hnRNP A1 dysfunction in oligodendrocytes was linked to MS, with altered expression levels correlating with demyelination and oligodendrocyte cell death.^92^

Beyond intrinsic dysfunction, autoantibodies targeting hnRNP A1 provide an additional layer of pathophysiological complexity in MS. These autoantibodies, which predominantly target the M9 domain responsible for nucleocytoplasmic shuttling, were detected in plasma and CSF samples of patients with MS.^93,94^ Once internalized via clathrin-mediated endocytosis, these autoantibodies promote hnRNP A1 mislocalization, stress granule formation, and decreased neurite length, a marker of neurodegeneration.^95,96^  *In vivo*, EAE mice treated with anti-hnRNP A1 antibodies exhibit heightened neurodegeneration and exacerbated clinical symptoms, further emphasizing the pathological relevance of these immune-mediated effects.^97^

Despite these insights, the exact mechanisms through which hnRNP A1 dysfunction contributes to MS pathogenesis remained unclear until recently. Recent transcriptomic and CLIP-sequencing analyses have provided new evidence, showing that hnRNP A1 mislocalization in progressive MS disrupts RNA metabolism.^98^ RNA sequencing identified approximately 550 differentially expressed genes in MS brains, 80% of which bind hnRNP A1 at the RNA level. Functional studies revealed that neuronal dysfunction of hnRNP A1 leads to neurite loss and splicing defects, demonstrating that hnRNP A1 dysregulation promotes neurodegeneration by disrupting A1-dependent RNA metabolic processes.^98^

Beyond hnRNP A1, other members of the hnRNP family have emerged as possible players in the complex pathogenesis of MS. For instance, antibodies against hnRNP B1 were detected in the CSF of patients with MS.^99^ Additionally, proteomic studies have revealed a striking reduction of approximately 90% in hnRNP K levels in human endothelial cells exposed to serum from patients with MS.^100^ Intriguingly, silencing hnRNP K expression during myelination results in impaired myelin basic protein synthesis, providing evidence for its involvement in myelination processes and its potential contribution to the pathophysiology of MS.^101^

Collectively, this body of evidence highlights the central role played by hnRNP proteins in the pathophysiology of MS, elucidating how their dysregulation drives neuronal dysfunction. Recent studies have further demonstrated that hnRNP-mediated disruptions in RNA metabolism are key drivers of neurodegeneration in MS, providing crucial insights into its pathogenic mechanisms and potential therapeutic strategies to combat the disease.

#### The frontotemporal lobar degeneration–amyotrophic lateral sclerosis spectrum

Amyotrophic lateral sclerosis (ALS), frontotemporal dementia (FTD) and frontotemporal lobar degeneration (FTLD) constitute a spectrum of neurodegenerative disorders, characterized by overlapping genetic, pathological and clinical features that range from motor neuron degeneration to cognitive and behavioural decline.^8^ Dysfunction in RNA-binding proteins, particularly hnRNPs such as transactive response DNA-binding protein-43 (TDP-43) and fused in sarcoma (FUS, also known as hnRNP P2) are known to contribute to neurodegeneration across the ALS-FTLD spectrum.^102,103^

Approximately 90% of ALS cases are sporadic, likely arising from complex interactions between genetic predisposition and ageing, whereas the remaining cases are familial, linked to mutations in over 30 genes, including many encoding RNA-binding proteins such as FUS.^104^ FUS is a ubiquitously expressed protein with essential functions in DNA repair, transcription and RNA splicing.^105-107^ Although primarily nuclear, FUS shuttles dynamically between cellular compartments, binding RNA in both the nucleus and cytoplasm.^107^ In neurons, it regulates critical processes such as dendritic spine maturation and excitatory synaptic transmission by modulating the RNA metabolism of key synaptic proteins, including GluA1 and SynGAP.^108,109^ ALS-associated mutations in FUS typically disrupt its NLS, leading to cytoplasmic mislocalization and aberrant accumulation in stress granules, which impairs their composition and function.^110-114^ A wealth of recent evidence supports a toxic gain-of-function mechanism in FUS-linked ALS caused by the cytoplasmic accumulation of the protein.^115-121^ Indeed, heterozygous *Fus* knock-in mouse models develop motor neuron degeneration, whereas *Fus* haploinsufficient knockout mice do not.^117^ Moreover, overexpression of wild-type FUS alone can trigger motor neuron degeneration, further reinforcing the pathogenic role played by cytoplasmic FUS accumulation in ALS.^118,119^ While cytoplasmic FUS is a well-established driver of motor neuron degeneration in ALS-FUS, its impact on synaptic function is less understood. Knock-in mouse models with cytoplasmic FUS accumulation reveal not only motor dysfunction, but also widespread behavioural abnormalities, cortical hyperexcitability and inhibitory synaptic disruptions.^120^ In a heterozygous knock-in model with an NLS deletion, mislocalized FUS accumulates at synapses, perturbing the regulation of key RNAs encoding GABAergic signalling components.^121^ These findings suggest that early disruptions in GABAergic synapses precede motor symptoms, potentially contributing to behavioural changes such as hyperactivity and social disinhibition. Notably, patient-derived neurons from individuals with ALS-FUS exhibit increased synaptic protein synthesis and functional dysregulation, further implicating synaptic dysfunction caused by dysfunctional FUS as an early pathological event in disease progression.^122^

TDP-43, a member of the hnRNP family, encoded by *TARDBP*, plays essential roles in transcription, RNA splicing and processing.^123,124^ In the ALS-FTLD spectrum, TDP-43 proteinopathy is a hallmark pathology, characterized by its mislocalization from the nucleus to the cytoplasm, leading to aggregate formation. This phenomenon is observed in 97% of ALS cases and in approximately half of patients with FTD.^102,125,126^ TDP-43-mediated neurodegeneration is driven by both loss-of-function and gain-of-function mechanisms, which act in tandem to disrupt neuronal homeostasis.^127^ The interplay between both mechanisms remains a subject of active investigation, with evidence supporting their concurrent involvement in neuronal degeneration.^127-129^ Loss-of-function occurs as nuclear depletion of TDP-43 disrupts pre-mRNA splicing, particularly by failing to repress cryptic exon inclusion in key neuronal genes such as *UNC13A* and *STMN2*, producing truncated, dysfunctional proteins.^130-132^ Beyond splicing defects, loss of nuclear TDP-43 is also associated with widespread transcriptomic changes and impaired chromatin structure.^133^ Conversely, gain-of-function mechanisms arise from cytoplasmic TDP-43 aggregates disrupting neuronal homeostasis. These aggregates alter gene expression patterns, impair local translation at neuromuscular junctions and contribute to mitochondrial dysfunction and neuroinflammation.^134-137^ Additionally, they sequester nuclear transport proteins, further exacerbating nuclear import deficits.^138,139^ Normally, TDP-43 autoregulates its own mRNA processing; however, in disease states, this feedback loop fails, increasing cytoplasmic *TARDBP* mRNA and promoting further aggregation.^140-142^ Meanwhile, clearance mechanisms fail to eliminate these aggregates, driving further protein accumulation and neuronal toxicity.^143-147^

While FUS and TDP-43 have emerged as central players in the pathogenesis of ALS-FTLD, increasing evidence also points to the involvement of additional hnRNPs in the molecular mechanisms underlying these diseases.^19^ For instance, mutations in the low-complexity domains of hnRNP A1 and A2/B1 have been linked to familial and sporadic ALS cases. These mutations predispose hnRNP A1 and hnRNP A2 to fibrillization, promote their excessive incorporation into stress granules and drive the formation of cytoplasmic inclusions.^148^ Notably, TDP-43 regulates hnRNP A1 splicing, and nuclear depletion of TDP-43 has been shown to drive the accumulation of an aggregation-prone isoform of hnRNP A1 in ALS.^149^ hnRNP A3, in contrast, has specifically been related to C9orf72-linked ALS, as it is mislocalized to the cytoplasm of patient motor neurons.^150^ Meanwhile, hnRNP Q has emerged as a modulator of TDP-43 toxicity, with increased levels observed in post-mortem ALS tissue.^151,152^ The TDP-43 binding partner hnRNP U has also been shown to influence TDP-43-induced neurotoxicity, as its overexpression attenuates TDP-43-driven neuronal cell death.^153^ Recent studies have also implicated hnRNP K in FTLD, particularly due to its mislocalization in the pyramidal neurons of the frontal cortex and the dentate nucleus of the cerebellum in affected individuals.^82,154^Interestingly, hnRNP M has recently been identified as a modulator of MATR3 toxicity, a DNA/RNA-binding protein that is mutated in familial ALS and whose pathological inclusions are also observed in sporadic cases of the disease.^102,155-158^

In summary, hnRNPs play a pivotal role in the pathogenesis of FTLD-ALS, where mutations and protein mislocalization drive widespread dysfunction in RNA metabolism and protein homeostasis. These insights not only deepen our understanding of disease progression but also position hnRNPs as compelling therapeutic targets within the ALS-FTLD spectrum.

### Heterogeneous nuclear ribonucleoproteins and neurodevelopmental disorders: decoding hnRNP H2

Neurodevelopmental disorders (NDDs) are multifaceted conditions that result in impairments in cognition, communication, behaviour and/or motor skills, typically associated with disruptions in essential neurodevelopmental processes.^159^ Intellectual disability, developmental delay, autism spectrum disorder and attention deficit hyperactivity disorder all fall under the NDD umbrella, affecting more than 3% of children worldwide.^159^ Among the hundreds of genes linked to NDDs, genes involved in RNA metabolic processing are shown to be enriched for *de novo* variants among probands with NDDs.^160^ As hnRNPs are essential for multiple steps of RNA metabolism in the brain, even subtle disruptions in their function can lead to significant neuronal consequences—a notion supported by the mounting evidence linking various hnRNP family members to NDDs.^161^ Overall, variants in 11 hnRNPs—*HNRNPC*, *HNRNPD*, *HNRNPG*, *HNRNPH1*, *HNRNPH2*, *HNRNPK*, *HNRNPR*, *SYNCRIP (HNRNPQ)*, *HNRNPU*, *HNRNPUL1* and *HNRNPUL2*—have been linked to NDDs, with developmental delay and intellectual disability among the most common features observed in affected individuals.^161^  ^,162^

As highlighted throughout this review, hnRNPs are essential regulators of neuronal RNA metabolism, orchestrating critical processes such as splicing, mRNA stabilization and translation. These fine-tuned mechanisms are fundamental for ensuring the precise expression of proteins that govern synaptic transmission, axonal growth and neuronal connectivity. Disruptions in these pathways can profoundly alter synaptic architecture and neuronal maturation, which are hallmark features of NDDs. Although hnRNP dysfunction has been studied extensively in the context of neurodegeneration, emerging evidence suggests a broader role in NDDs. hnRNPs are increasingly being recognized as central modulators of synaptic connectivity, neuronal differentiation and circuit integrity, highlighting their fundamental role in shaping the developing brain. Although the precise mechanisms remain to be fully elucidated, mutations affecting hnRNP function may disrupt these tightly regulated processes, leading to alterations in both the timing and progression of critical neurodevelopmental events.

A deeper mechanistic understanding of these pathways will not only provide critical insights into how hnRNP dysregulation contributes to NDDs but also refine our understanding of their roles in neuronal development. For example, recent investigations into *HNRNPH2*-related NDD have opened the door for new studies on how this protein may orchestrate fundamental neurodevelopmental processes, as discussed in the next sections.

#### The hnRNP F/H subfamily: insights into hnRNP H2

hnRNP H2 is a 449 amino acid RNA-binding protein encoded by *HNRNPH2* on chromosome Xq22.1.^163^ It belongs to the hnRNP F/H subfamily, which also includes hnRNPs F, H1, H3 and G-rich sequence factor 1 (GRSF1). hnRNP F/H proteins play crucial roles in RNA metabolism, primarily as regulators of alternative splicing, polyadenylation and other essential steps in RNA processing and transport.^164^ Like other F/H members, hnRNP H2 contains three qRRMs (which lack the RNP consensus sequences and feature an extra β_3_´ loop compared to classical RRMs), two glycine-rich domains and an NLS.^2^ This PY-NLS mediates the nuclear import of hnRNP H2 by interacting with the transport receptor karyopherin-β2 (Kapβ2), a mechanism conserved among its closely related counterparts.^165^ Owing to their structural similarities, the role of hnRNP H2 in RNA metabolism is often discussed alongside that of hnRNP H1, as demonstrated by their collaborative regulation of *U11-48K* pre-mRNA stability, which encodes a component of the minor spliceosome complex.^166^ While hnRNP H1 is well characterized for its involvement in splicing, mRNA stability and polyadenylation, the specific contributions of hnRNP H2 to these mechanisms remain less explored.^167^ Nonetheless, it was shown that hnRNP H2 participates in alternative splicing regulation, including the regulation of the thymidine phosphorylase (TP) pre-mRNA, where it promotes intron retention and suppresses translation.^168^ Additionally, hnRNP H2 contributes to pre-mRNA cleavage and polyadenylation, reinforcing its role in post-transcriptional gene regulation.^169,170^ Although these findings are significant, the functional distinctions between hnRNP H2 and its closely related counterparts remain unclear, warranting further investigation into its mechanistic contributions to RNA metabolism.

#### The emerging role of hnRNP H2 in neuronal RNA metabolism

**Figure 3:**
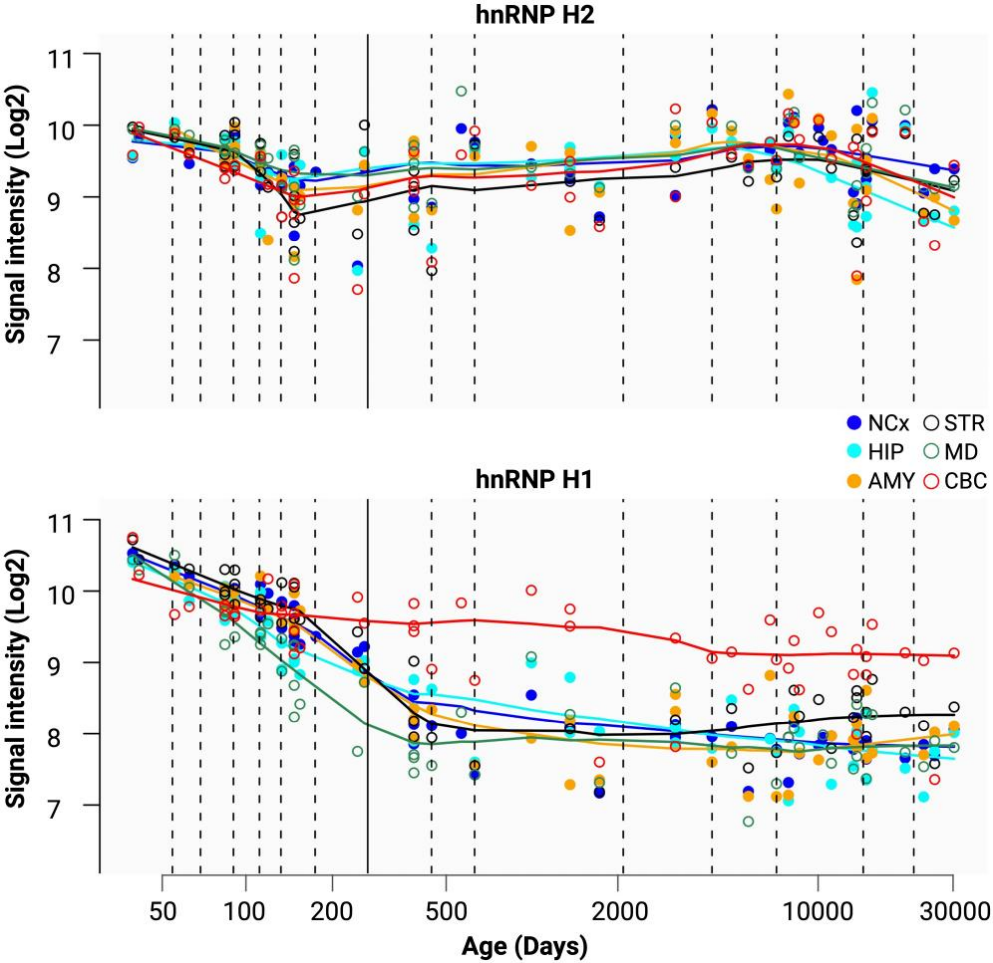
**Expression of *HNRNPH2* and *HNRNPH1* in the human brain.**  *HNRNPH2* and *HNRNPH1* are highly expressed across the human brain from early development through late adulthood. Despite their structural homology, these proteins display different spatiotemporal expression patterns, suggesting specialized roles in orchestrating neurodevelopmental processes and sustaining neural function throughout the lifespan. Adapted from the Human Brain Transcriptome (https://hbatlas.org/), based on data from references.^175-177^ AMY = amygdala; CBC = cerebellar cortex; HIP = hippocampus; MD = mediodorsal nucleus of the thalamus; NCx = neocortex; STR = striatum.

Recent evidence suggests that hnRNP H2 plays a critical role in neuronal differentiation, primarily through the regulation of telomere repeat-binding factor 2 (TRF2) pre-mRNA splicing. Alternative splicing of exon 7 in TRF2 generates a truncated isoform, TRF2-S, which lacks both the DNA-binding domain and the NLS. Consequently, TRF2-S is retained in the cytoplasm, where it promotes neuronal differentiation.^171^ hnRNP H2 directly binds to TRF2 pre-mRNA, repressing the alternative 5′ splice site and promoting the inclusion of exon 7, thereby limiting the production of TRF2-S.^172^ Interestingly, deletion of hnRNP H2 selectively accelerates neuronal differentiation, suggesting that hnRNP H2 functions as a negative regulator of this process through splicing-mediated mechanisms.^172^ Beyond this, hnRNP H2 also plays a broader role in neuronal RNA metabolism. Simultaneous depletion of hnRNP H1 and H2 in rat dorsal root ganglion neurons reduces axonal mRNA translation, implicating these proteins in local protein synthesis regulation.^173^ Additionally, knockdown of both proteins impairs axonal branching, suggesting a cooperative role in shaping neuronal morphology.^173^ Further supporting its involvement in neuronal processes, hnRNP H2 expression levels were shown to decrease in the cerebral cortex of mice chronically exposed to nicotine, indicating a potential role in neural adaptation to environmental stimuli.^174^ These findings highlight hnRNP H2 as a crucial regulator of neuronal RNA metabolism, influencing processes such as differentiation, intra-axonal translation, and neuronal morphology. Human brain transcriptome shows that *HNRNPH2* is expressed throughout development and across the lifespan (Fig. 3), pointing to its significant, yet largely unexplored, role in brain development and function.^175-177^ As research into the function of hnRNP H2 in the nervous system progresses, further studies will be essential to uncover its precise mechanisms and broader implications for neural development and plasticity.

#### The genetic and molecular basis of hnRNP H2-related neurodevelopmental disorder

**Figure 4:**
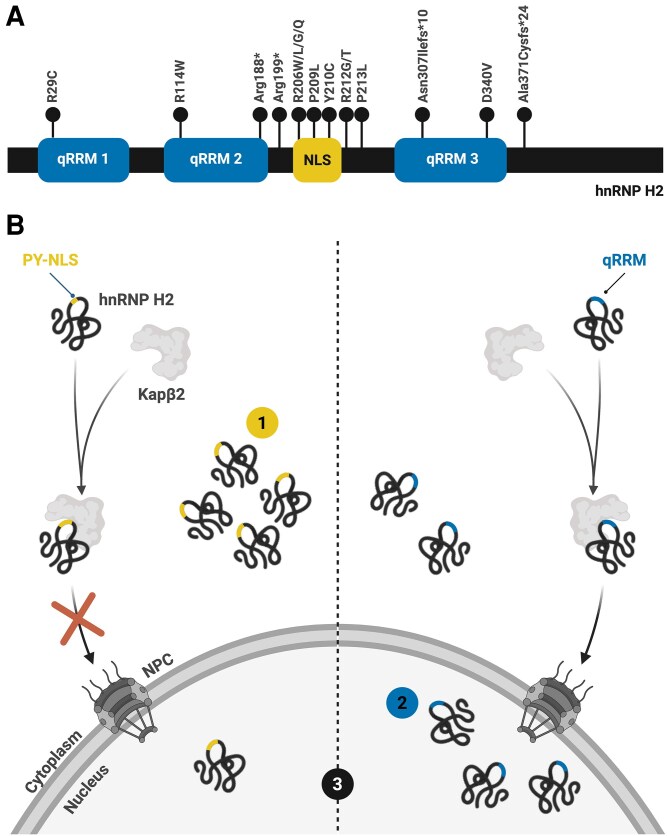
***HNRNPH2*-related neurodevelopmental disorder.**  *HNRNPH2*-related neurodevelopmental disorder (*HNRNPH2*-RNDD) is a rare X-linked syndrome marked by developmental and neurological impairments. (**A**) The genetic aetiology of *HNRNPH2*-RNDD was elucidated through the identification of *de novo* pathogenic variants in the *HNRNPH2* gene, with mutations predominantly clustering within the nuclear localization signal (NLS) and qRRMs of the encoded protein. While initially presumed to be male-lethal, emerging reports of affected males have challenged this paradigm, revealing a broader and more heterogeneous clinical spectrum than previously appreciated.^161,163,179-184^ (**B**) Mechanistically, *HNRNPH2*-RNDD reflects a complex interplay between impaired protein localization and disrupted RNA processing. NLS mutations compromise nuclear import, resulting in cytoplasmic accumulation of hnRNP H2 (1), while the R114W mutation within the qRRM domain disrupts essential interactions with nuclear splicing regulators (2). Both mutation types converge on aberrant RNA splicing and gene expression (3), potentially implicating defective neuronal RNA metabolism as a central driver of the neurodevelopmental pathology.^185,186^ Created in BioRender. Costa, R. (2026) https://BioRender.com/jzpixiq. Kapβ2 = karyopherin-β2; NPC = nuclear pore complex; PY-NLS = proline-tyrosine nuclear localization signal; qRRM1 = quasi-RNA recognition motif 1; qRRM2 = quasi-RNA recognition motif 2; qRRM3 = quasi-RNA recognition motif 3. * premature stop codon.

In 2016, Bain and colleagues^163^ identified pathogenic *HNRNPH2* variants in six unrelated females, establishing these mutations as the cause of an X-linked NDD. Five patients carried missense mutations at amino acid 206 (R206W or R206Q), while a sixth had a P209L variant, all within the NLS of hnRNP H2.^163^ This discovery led to the classification of *HNRNPH2*-related NDD (*HNRNPH2*-RNDD), also known as intellectual developmental disorder, X-linked syndromic, Bain type (OMIM 300986). The disorder is characterized by developmental delay, intellectual disability, language and motor impairments, growth abnormalities and musculoskeletal disturbances.^178^ Although most *HNRNPH2* mutations arise *de novo*, inherited cases, such as those involving germline mosaicism or skewed X-inactivation in carrier mothers, have also been described.^179^  ^,180^ To date, approximately 50 individuals with *HNRNPH2*-RNDD have been reported, with mutations primarily clustering in the NLS and qRRMs of hnRNP H2 (Fig. 4A).^161,163,178-184^ The pathogenic mechanisms underlying *HNRNPH2*-RNDD remain unclear; however, emerging evidence suggests distinct functional consequences depending on the mutation site (Fig. 4B). Variants in the NLS (e.g. R206W and P209L) disrupt nuclear import, leading to cytoplasmic accumulation of hnRNP H2, where it may be incorporated into RNA-protein stress granules.^184-186^ Meanwhile, the R114W mutation in the qRRM domain alters alternative splicing, gene expression and the interaction of hnRNP H2 with nuclear splicing regulators.^184^ Mouse models of *HNRNPH2*-RNDD exhibit phenotypes resembling those observed in human patients, including craniofacial abnormalities, motor deficits, seizures and cognitive impairments.^186^ Interestingly, *HNRNPH2* knockout mice do not display these symptoms, likely due to compensatory upregulation of *HNRNPH1*, a mechanism absent in mutant animals.^186^ This suggests that while hnRNP H1 can functionally compensate for hnRNP H2 loss, pathogenic *HNRNPH2* mutations interfere with this compensation. Consequently, two possible disease mechanisms have been proposed: a toxic gain-of-function effect or a loss of hnRNP H2 function that disrupts hnRNP H1-mediated compensation.^186^ Despite these advances, the precise neuronal consequences of *HNRNPH2* mutations and the functional interplay between hnRNP H2 and hnRNP H1 remain poorly understood. Further research will be critical for clarifying these mechanisms and guiding the development of targeted therapies for *HNRNPH2*-RNDD.

## Concluding remarks and future perspectives

The advances in hnRNP biology have highlighted the central role of these multifaceted proteins as regulators of RNA metabolism, coordinating processes such as alternative splicing, mRNA stabilization and translation that are essential for neuronal function. Disruption of these roles compromises neuronal integrity and contributes to a broad spectrum of neurological disorders. This evolving insight has reshaped our understanding of hnRNPs; rather than being viewed merely as disease markers, they are now recognized as dynamic therapeutic targets capable of restoring cellular function and mitigating neurological dysfunction.

Recent advances have provided compelling evidence that modulating hnRNP expression yields significant therapeutic benefits. For instance, downregulation of neuronal PTBP1 alleviates motor deficits in Parkinson’s disease models, while overexpression of the long isoform of hnRNP DL improves cognitive function in Alzheimer’s models.^187,188^ Furthermore, oestradiol treatment has been shown to upregulate hnRNP A1 expression and promote APP695 mRNA formation, which is associated with reduced amyloid-β production.^70^ Moreover, elevated hnRNP A1 further mitigates amyloid-β toxicity through enhanced transcriptional regulation of hexokinase I (HK1) mRNA.^76^ Beyond directly manipulating hnRNP levels, targeting the downstream pathogenic consequences of hnRNP dysfunction may offer a complementary therapeutic avenue. For example, in ALS, reducing RNA m^6^A modification levels has shown potential in inhibiting toxic cytoplasmic protein aggregates in cells expressing mutant FUS,^189^ whereas the targeted introduction of human DNA ligase 1 to repair mitochondrial DNA damage in FUS mutant cells further illustrates the versatility of these strategies.^190^

Innovative therapies, including antisense oligonucleotides (ASOs), have also emerged as promising interventions for hnRNP-related disorders. In ALS-FUS mouse models, an ASO targeting the FUS transcript not only reduced FUS protein levels but also delayed motor neuron degeneration, with subsequent human studies demonstrating a marked decrease in pathological FUS aggregates in both the spinal cord and motor cortex.^191^ In parallel, a novel precision medicine strategy—TDP-REG—leverages the aberrant inclusion of cryptic exons caused by TDP-43 loss-of-function to drive therapeutic transgene expression specifically in affected cells. TDP-REG vectors encoding a TDP-43 fusion protein effectively rescued critical pathological cryptic splicing events linked to ALS, paving the way for precision therapies for this and other TDP-43-related disorders.^192^

Collectively, these findings mark a pivotal shift in the therapeutic landscape of hnRNP-related disorders. As our understanding of the molecular underpinnings of hnRNP function and dysfunction deepens, the potential to develop highly targeted interventions, whether by modulating hnRNP expression, correcting their mislocalization or reshaping their RNA interactome, becomes increasingly tangible. Moving forward, a critical priority will be the in-depth characterization of hnRNP-regulated neuronal RNA networks and their perturbations in disease contexts (see the ‘Outstanding questions’ section). Such knowledge will not only enhance our mechanistic insights but may also contribute to the development of rationally designed interventions to combat a wide array of neurodegenerative and neurodevelopmental disorders rooted in hnRNP dysfunction.

## Outstanding questions

Recent single-cell transcriptomic and spatial profiling studies have revealed that hnRNP expression is both cell-type- and region-specific across the brain. In the human and murine cortex, hnRNPs are broadly expressed during development but are particularly enriched in cortical progenitors compared to postmitotic neurons, suggesting specialized regulatory roles during neuronal maturation.^8^ Together, these insights point to critical questions. Do hnRNP proteins exert cell-type–specific and region-specific RNA regulatory functions across diverse neuronal populations in the brain? How does this spatial heterogeneity influence neuronal function and susceptibility to disease?

As hnRNPs interact with other RNA-binding proteins and RNA-modifying enzymes, forming networks that coordinate RNA metabolism and function, how do these interactions shape the neuronal transcriptome under both physiological and pathological conditions?

hnRNPs engage in auto- and cross-regulatory mechanisms to modulate their own expression and that of other family members. Does this regulatory network maintain dosage and temporal control of hnRNP activity in neurons? How does its disruption contribute to disease?

Mislocalization of hnRNPs is consistently observed in several neurodegenerative disorders, and recent evidence suggests similar phenomena in neurodevelopmental conditions. These shared patterns of mislocalization highlight a potential convergent mechanism across distinct brain pathologies, raising key questions about the molecular processes that govern hnRNP trafficking between the nucleus and cytoplasm. Can restoring their proper subcellular localization reverse downstream RNA dysregulation and neuronal damage?

hnRNP dysfunction in multiple brain disorders leads to altered RNA splicing and widespread changes in gene expression. These disruptions raise fundamental questions about the underlying mechanisms and therapeutic implications of hnRNP-related pathology. Are the neurological consequences of hnRNP dysfunction driven by the cumulative disruption of hundreds of RNA processing events over time, or can misregulation of a few key transcripts initiate disease? Is correcting a subset of critical RNA targets sufficient to restore neuronal homeostasis? Does the spectrum of RNA targets and pathways affected by hnRNP dysfunction shift during disease progression, and could these dynamic changes influence the timing and efficacy of RNA-targeted therapies?

Conserved structural motifs and overlapping RNA-binding properties, suggest a potential functional redundancy among hnRNP family members. Indeed, emerging evidence indicates that loss of specific hnRNPs can trigger compensatory upregulation of close paralogues.^186^ This raises key questions. What are the physiological roles and regulatory limits of these compensatory mechanisms, and could they be leveraged therapeutically to mitigate hnRNP-related dysfunction?
